# Gene Expression Profiling for Differential Diagnosis of Liver Metastases: A Multicenter, Retrospective Cohort Study

**DOI:** 10.3389/fonc.2021.725988

**Published:** 2021-09-22

**Authors:** Qifeng Wang, Fen Li, Qingming Jiang, Yifeng Sun, Qiong Liao, Huimin An, Yunzhu Li, Zhenyu Li, Lifang Fan, Fang Guo, Qinghua Xu, Yixin Wo, Wanli Ren, Junqiu Yue, Bin Meng, Weiping Liu, Xiaoyan Zhou

**Affiliations:** ^1^Department of Pathology, Fudan University Shanghai Cancer Center, Shanghai, China; ^2^Department of Oncology, Shanghai Medical College, Fudan University, Shanghai, China; ^3^Institute of Pathology, Fudan University, Shanghai, China; ^4^The Cancer of Unknown Primary Group of Pathology Committee, Chinese Research Hospital Association, Shanghai, China; ^5^Department of Pathology, Chengdu Second People’s Hospital, Chengdu, China; ^6^Department of Pathology, Chongqing University Cancer Hospital, Chongqing, China; ^7^The Canhelp Genomics Research Center, Canhelp Genomics Co., Ltd., Hangzhou, China; ^8^Department of Pathology, Sichuan Cancer Hospital, Chengdu, China; ^9^Department of Pathology, Sir Run Run Shaw Hospital, College of Medicine, Zhejiang University, Hangzhou, China; ^10^Department of Pathology, Hubei Cancer Hospital, Tongji Medical College, Huazhong University of Science and Technology, Wuhan, China; ^11^The Institute of Machine Learning and Systems Biology, College of Electronics and Information Engineering, Tongji University, Shanghai, China; ^12^Xuzhou Engineering Research Center of Medical Genetics and Transformation, Department of Genetics, Xuzhou Medical University, Xuzhou, China; ^13^Department of Pathology, National Clinical Research Center of Cancer, Key Laboratory of Cancer Prevention and Therapy, Tianjin’s Clinical Research Center for Cancer, Tianjin Medical University Cancer Institute and Hospital, Tianjin, China; ^14^Department of Pathology, West China Hospital, Sichuan University, Chengdu, China

**Keywords:** liver metastasis, tissue of origin, gene expression profiling, real-time PCR, tumor classification

## Abstract

**Background:**

Liver metastases (LM) are the most common tumors encountered in the liver and continue to be a significant cause of morbidity and mortality. Identification of the primary tumor of any LM is crucial for the implementation of effective and tailored treatment approaches, which still represents a difficult problem in clinical practice.

**Methods:**

The resection or biopsy specimens and associated clinicopathologic data were archived from seven independent centers between January 2017 and December 2020. The primary tumor sites of liver tumors were verified through evaluation of available medical records, pathological and imaging information. The performance of a 90-gene expression assay for the determination of the site of tumor origin was assessed.

**Result:**

A total of 130 LM covering 15 tumor types and 16 primary liver tumor specimens that met all quality control criteria were analyzed by the 90-gene expression assay. Among 130 LM cases, tumors were most frequently located in the colorectum, ovary and breast. Overall, the analysis of the 90-gene signature showed 93.1% and 100% agreement rates with the reference diagnosis in LM and primary liver tumor, respectively. For the common primary tumor types, the concordance rate was 100%, 95.7%, 100%, 93.8%, 87.5% for classifying the LM from the ovary, colorectum, breast, neuroendocrine, and pancreas, respectively.

**Conclusion:**

The overall accuracy of 93.8% demonstrates encouraging performance of the 90-gene expression assay in identifying the primary sites of liver tumors. Future incorporation of the 90-gene expression assay in clinical diagnosis will aid oncologists in applying precise treatments, leading to improved care and outcomes for LM patients.

## Introduction

Liver metastases (LM) are tumors that have propagated to the liver from tumors originating from other parts of the body. Due to the venous blood returning from the gastrointestinal system through portal vein circulation, gastrointestinal tract tumors are more likely to metastasize to the liver (1). Besides, the liver microenvironment also plays a significant role in the development of hepatic metastasis. Numerous studies have shown that both the acellular such as extracellular matrix proteins (i.e. collagen) and the cellular components of the liver such as Kupffer cells, hepatic stellate cells and liver sinusoidal endothelial cells contribute to the metastatic ability of tumors of different origins (2). According to the statistical data in the Surveillance, Epidemiology and End Results (SEER) database, 5.14% to 6.46% of cancer patients are diagnosed with synchronal LM at the time of primary cancer diagnosis (3, 4). Of note, during the course of the cancer disease, up to 50% of patients with various tumor types will either present with or develop LM (1). The most common tumor that spreads to the liver is breast cancer for younger women and colorectal cancers for younger men (3). In the current era, several studies investigated that the incidence rate of cancer of unknown primary (CUP) is currently decreasing and reaches 1-2% (5). Liver CUP is the most common CUP subgroup (30–40%) and has the most dismal prognosis with median overall survival (OS) of 1–2 months and one-year OS of 5–12% (6).

The prognosis of LM varies to tumor types. LM originated from small intestine cancer shows the best prognosis, followed by testis cancer and breast cancer (4). Traditionally, the treatment approaches were established according to the primary tumor of LM. For example, resection can be usually performed in patients with colorectal liver metastases (CLM) and neuroendocrine tumor liver metastases (NETLM), but it may be not appropriate for patients with LM from pancreatic cancer, esophageal cancer, melanoma and adrenocortical cancer (1). In addition, different tumor types carry specific genetic alterations, genomic feature analysis which could provide precise and pertinent clinical details for disease management. For CLM patients, information on the mutation status of oncogenes such as *BRAF, NRAS*, and *KRAS* as well as analysis of microsatellite instability (MSI) status have led to precise therapy and prognostic stratification (7). Therefore, identification of the primary tumor of any LM is pivotal for the implementation of valid and tailored treatment options, which still acts as a troublesome problem in the clinical setting. In most cases, metastatic tumors with representative histological features similar to the primary lesion can be correctly distinguished with hematoxylin-eosin (H&E) staining and immunohistochemistry (IHC) (8). However, the distinction between intrahepatic cholangiocarcinoma and metastatic adenocarcinoma is frequently challenging owing to the overlapping phenotypic profiles (9).

Over the last decades, molecular profiling has been under speed development for predicting tumor site of origins in CUP patients (10, 11). According to the tumor origin, specific gene expression profiling has been well recognized in most tumor types, which reflects the different expression profilings in their normal tissues of origin. Differences in gene expression pattern thus allow distinction between various solid tumors and provide a valuable method for diagnosis of the tissue of origin in CUP patients. Recently, our group has developed a 90-gene expression assay for the classification of 21 common tumor types which represent approximately 95% of the incident solid tumors that are known to produce distant metastases. In a retrospective cohort of 609 clinical specimens, the 90-gene expression assay illustrated an overall accuracy of 90.4% for primary tumors and 89.2% for metastatic tumors. Furthermore, in a real-world cohort of 141 CUP patients, the gene expression assay was able to provide instructive predictions of primary tumors in 71.6% of patients (101 of 141). These findings suggest that the 90-gene expression assay could efficiently identify the primary site for a broad spectrum of tumor types and support its diagnostic utility of molecular classification in difficult-to-diagnose metastatic tumors (12). Recently, Wang et al. performed the 90-gene expression assay for the differential diagnosis of metastatic triple-negative breast cancer (TNBC) (13). This assay correctly identified 97.6% of TNBC lymph node metastases (41 of 42) and 96.8% of distant metastatic tumors (30 of 31). Zheng et al. investigated the potential utility of the 90-gene expression assay in diagnosing the tumor origin of brain tumors (14). The molecular assay illustrated 100% accuracy for discriminating primary brain tumors from brain metastases and accurately predicted primary sites for 89% of brain metastases (39 of 44).

In the present study, we conducted a multi-center retrospective study based on seven cancer centers in China to assess the performance of the 90-gene expression assay and explore its potential diagnostic utility for LM.

## Material and Methods

### Patient Enrollment and Specimen Acquisition

The study protocol was approved by the institutional review board of Fudan University Shanghai Cancer Center (FUSCC, Shanghai, China), West China Hospital Sichuan University (WCHSU, Chengdu, Sichuan, China), Sichuan Cancer Hospital (SCH, Chengdu, Sichuan, China), Chongqing Cancer Hospital (CCH, Chongqing, China), Tianjin Medical University Cancer Institute & Hospital (TMUCIH, Tianjin, China), Sir Run Run Shaw Hospital (SRRSH, Hangzhou, Zhejiang, China) and Hubei Cancer Hospital (HCH, Wuhan, Hubei, China). Between January 2017 and December 2020, a total of 156 surgical or biopsy specimens from the liver and associated clinicopathologic data were archived from seven independent centers, and 146 cases (130 LM and 16 primary liver tumors) that met all criteria were enrolled in the present study. Biopsy samples were obtained by needle core biopsy (NCB) or fine-needle aspiration (FNA), using either transabdominal ultrasound or computed tomography (CT) guidance. The inclusion criteria for all specimens were the following: (1) formalin-fixed, paraffin-embedded (FFPE) tumor tissues, (2) the primary tumor sites were verified through evaluation of available medical records, pathological and imaging information. The reference diagnosis of primary tumor was conform to the 21 tumor types of the assay (**Supplementary Table 1**) (12), (3) at least 60% tumor cell content, and (4) less than 40% necrosis based on the H&E staining evaluation.

### Sample Preparation and RNA Isolation

Five to fifteen 5μm unstained sections were freshly cut for gene expression analysis. The FFPE tissue samples were centralized and the H&E-stained slide of each case had been reviewed for evaluation of the percentage of tumor cells and necrotic areas by two senior pathologists from FUSCC (QF W and XY Z). The regions of tumor tissue were marked on the H&E-stained slides and macro-dissected manually for tumor cells enrichment. Total RNA isolation and gene expression profiling were performed at the Canhelp Genomic Reference Laboratory (Hangzhou, China). Total RNA was extracted using a FFPE Total RNA Isolation Kit (Canhelp Genomics Co., Ltd., Hangzhou, China) according to the protocols. Briefly, FFPE tissue was deparaffinized, followed by digestion, DNase treatment and total RNA elution. The concentration of total RNA was measured by spectrophotometer at 260-nm absorbance, and the purity was quantified using A260/A280 ratio. RNA samples with A260/A280 ratios between 1.7 and 2.1 were enrolled in this study.

### Gene Expression Profiling Analysis

After performing reverse transcription on isolated total RNA (2ug per specimen) using a High-Capacity cDNA Reverse Transcription Kit with RNase Inhibitor (Applied Biosystems, Foster City, CA), the 90-gene real-time PCR (RT-PCR) assay (Canhelp Genomics, Hangzhou, China). was analyzed with a 7500 Real-Time PCR system (Applied Biosystems) to analyze cancer-specific gene expression profiles. The RT-PCR program was initiated at 95°C for 10 minutes, followed by 40 cycles at 95°C for 15 seconds and 60°C for 1 minute. To correct for input variation, for each sample, cycle threshold (Ct) measurements of target genes were normalized to multiple reference genes. For samples with the Ct values of reference genes greater than 38 were excluded. The 90-genes expression data of valid samples were provided in **Supplementary Table 2**.

### 90-Gene Classifier for Tumor Classification: Algorithm Development and Data Analysis

Initially, the cancer-specific gene markers were identified based on a pan-cancer transcriptome database comprising 5434 specimens representing 21 tumor types (15). The database included both primary and metastatic tumors and well-differentiated to undifferentiated tumors. The SVM-RFE (Support Vector Machine-Recursive Feature Elimination) machine learning algorithm was used to select the Top-10 most predictive genes for each of the 21 tumor types. After removing redundant genes, a list of 90 genes specific to 21 tumor types was identified. Details of the 90-gene list were provided in **Supplementary Table 3**. Then, an SVM linear model was trained using the whole pan-cancer transcriptome database to form a multiclass classification algorithm (“90-gene classifier”).

Mathematically, the 90-gene classifier creates a hyperplane for each tumor type in a 90-dimensional space. For an unknown test sample, the algorithm calculates its 90 genes’ expression values, projects it to the 90-dimensional space, and estimates the distance of the test sample to each of 21 hyperplanes. The position of the test sample relative to the hyperplane determines its membership in one or the other class (e.g., ‘‘breast cancer’’ *vs*. “not breast cancer’’). Furthermore, the confidence of the test sample belongs to a tumor type is proportional to the distance of the test sample from the corresponding hyperplane. The far the distance, the higher the confidence. Then, the distances of the test sample from each of the 21 hyperplanes were compared and transformed to the similarity scores with the Platt Scaling formula (16). Intuitively, the similarity scores reflect how much the gene expression pattern of the test sample is similar to the global gene expression pattern of the indicated tumor type. The similarity scores were probability-based, with a reported range from 0 to 100, and all 21 scores sum to 100. The tumor type with the highest similarity score was defined as the predicted tumor type by the 90-gene classifier. An example was shown in **Supplementary Figure 1**. The primary site with the highest similarity score is gastroesophagus, thus indicating the most likely tissue of origin is gastroesophagus.

### Statistical Analysis

All statistical analysis was performed using the R software (version 3.6.1) and packages from the Bioconductor project (version 3.9). The hierarchical clustering of clinical specimens based on the gene expression pattern was performed using “pheatmap” package (version 1.0.12). The average linkage hierarchical clustering method was performed where the metric of similarity was Pearson’s correlation between every pair of samples. The receiver operating characteristic (ROC) curves were estimated using “multiROC” package (version 1.1.1). The gene expression assay performance was assessed by calculating the area under curve (AUC) for each tumor type and aggregation across all tumor types. For multi-class evaluation, the AUC for all tumor types was calculated through a micro-averaging approach, which stacked all tumor types together, thus converting the multiclass classification into binary classification. The micro-averaging approach further considered the contributions of different tumor types and weight metrics toward the largest type when some tumor types have more instances than others. P-value was computed two-sided and considered as statistically significant if p-value < 0.05.

## Results

### Patients and Samples

**Figure 1:**
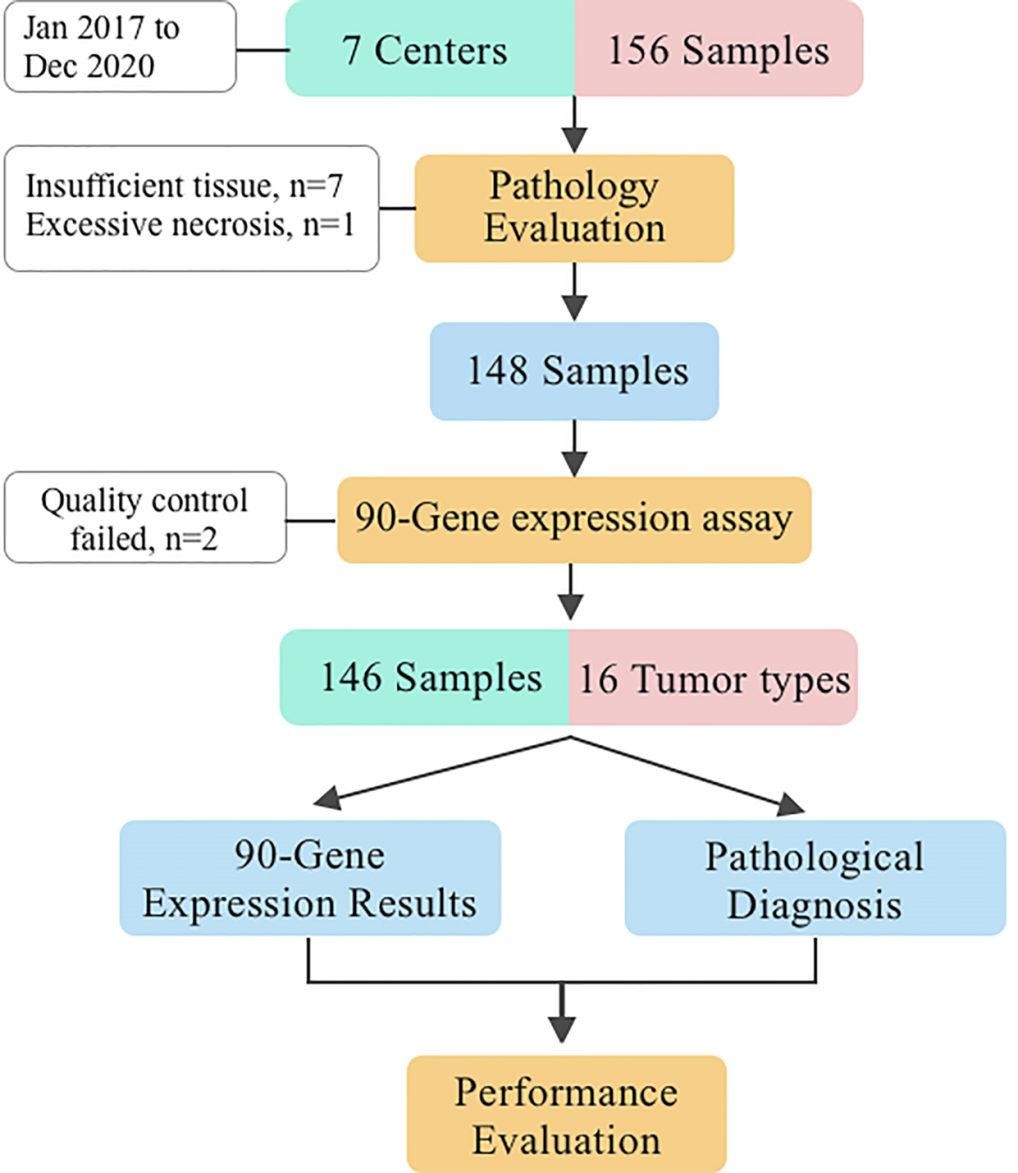
Study design.

Initially, 156 FFPE specimens covering 16 primary tumor types were collected in the present study, 148 had successful histologic quality control and 146 of these samples passed the RT-PCR quality control. More specifically, seven specimens were excluded because of less than 60% tumor cell content, one because of more than 40% necrosis, and two because of RT-PCR quality control failures. Finally, 130 LM and 16 primary liver tumor specimens met all quality control criteria and were successfully analyzed by the 90-gene expression assay. The overall study design is presented in **Figure 1**. The sample enrollment of seven center hospitals was shown in **Supplementary Table 4**.

The demographics and clinical characteristics of the cohort are provided in **Table 1**. The cohort included 63 males and 83 females, with a median age of 57.5 years old (range 14-83). All specimens were taken from the liver, of which 36 were biopsy samples and 110 were resection samples. The origin of LM came from 15 primary sites and the most common tissue of origin were colorectum (n=23) and ovary (n=23), followed by breast (n=19), neuroendocrine (n=16), pancreas (n=16) and gastroesophagus (n=10). Other relatively rare tumor types comprising melanoma (n=4), cervix (n=4), lung (n=3), adrenal (n=3), germ cell (n=2), head&neck (n=2), sarcoma (n=2), kidney (n=2) and urinary (n=1) were also included. Among 146 samples, the degree of differentiation of 97 cases was defined, 26 (26.8%) cases were well-differentiated, whereas 71 (73.2%) cases were poorly differentiated.

**Table 1:** The demographics and clinical characteristics of the cohort.

Characteristic	Number of specimens (N = 146)	Percentage (%)
**Gender**		
Male	63	43.2
Female	83	56.8
**Age**		
Median	57.5	
Range	14-83	
**Tumor types**		
Liver	16	11.0
Colorectum	23	15.8
Ovary	23	15.8
Breast	19	13.0
Neuroendocrine	16	11.0
Pancreas	16	11.0
Gastroesophagus	10	6.8
Melanoma	4	2.7
Cervix	4	2.7
Lung	3	2.1
Adrenal	3	2.1
Germ cell	2	1.4
Head&neck	2	1.4
Sarcoma	2	1.4
Kidney	2	1.4
Urinary	1	0.7
**Histological Subtype**		
Adenocarcinoma	114	78.1
Neuroendocrine	16	11.0
Squamous cell carcinoma	10	6.8
Melanoma	4	2.7
Sarcoma	2	1.4
**Degree of differentiation^1^**		
Well-differentiated	26	26.8
Poorly differentiated	71	73.2

^1^The degree of differentiation of 49 specimens was undefined.

### Performance of the 90-Gene Expression Assay in Liver Tumors

**Figure 2:**
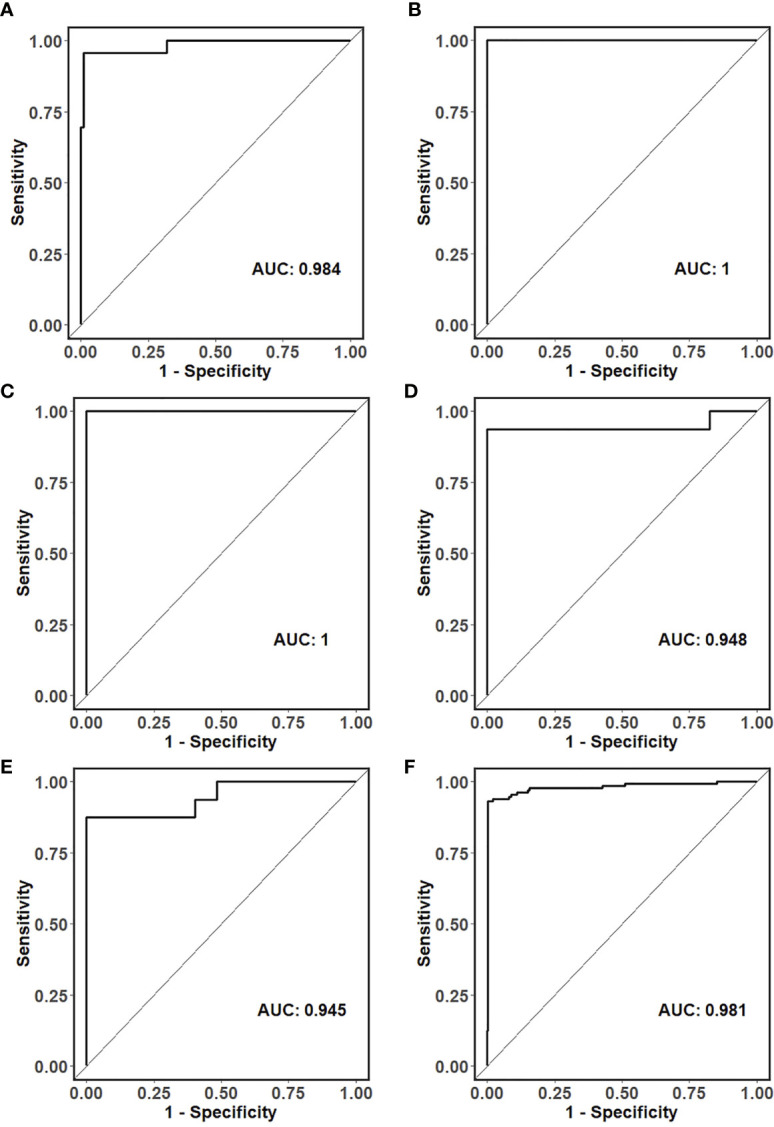
ROC curves for the classification of the tissue of origin in **(A)** colorectal, **(B)** ovarian, **(C)** breast, **(D)** neuroendocrine, **(E)** pancreatic and **(F)** all liver metastatic tumors.

For primary liver tumors, the 90-gene expression assay correctly classified all 16 samples showing a 100% accuracy. For 130 LM cases, the 90-gene expression assay achieved a 93.1% (121/130, 95% CI: 0.87-0.97) accuracy by comparing the predicted tumor types with the reference diagnosis. The AUC of the Top-5 common tumor types ranged from 0.945 to 1 (**Figures 2A–E**), and the weighted AUC for all tumor types reached 0.981 (**Figure 2F**). As shown in **Table 2**, the sensitivities of the 90-gene expression assay are variable, ranging from 50% (head&neck) to 100% (ovary, breast, melanoma, etc.).

**Table 2:** The performance of the 90-gene expression assay in liver metastases.

Tumor type	Number of samples	Correctly classified samples by the gene expression assay	Sensitivity (%)
Ovary	23	23	100
Colorectum	23	22	95.7
Breast	19	19	100
Neuroendocrine	16	15	93.8
Pancreas	16	14	87.5
Gastroesophagus	10	7	70.0
Melanoma	4	4	100
Cervix	4	4	100
Lung	3	2	66.7
Adrenal	3	3	100
Germ Cell	2	2	100
Head&neck	2	1	50.0
Sarcoma	2	2	100
Kidney	2	2	100
Urinary	1	1	100
**Total**	130	121	93.1

Of the 146 specimens, 26 were well or moderately differentiated tumors, 71 were poorly or undifferentiated tumors, and 49 were not specified. More specifically, the classification accuracy was 96.2% (25 of 26) for well or moderately differentiated tumors and 88.7% (63 of 71) for poorly or undifferentiated tumors, with no statistically notable difference (p = 0.47). In addition, the present study enrolled 36 biopsy specimens and 110 resection specimens. The overall accuracy of 90-gene expression assay showed no significant difference between biopsy and resection groups, (88.9% and 95.4%, respectively, p value equals 0.31).

In subgroup analysis, the neuroendocrine tumors were originated from pancreas (n=5), gallbladder (n=2), thyroid (n=2), ovary (n=1), esophagus (n=1), lung (n=1) and undefined (n=4), with an overall accuracy of 93.8% (15/16). For ten cases of squamous cell carcinoma, their origins were composed of the cervix (n=4), gastroesophagus (n=3), head&neck (n=2), and lung (n=1). The 90-gene signature correctly classified the tissue of origin in 7 of 10 cases (70%).

**Figure 3:**
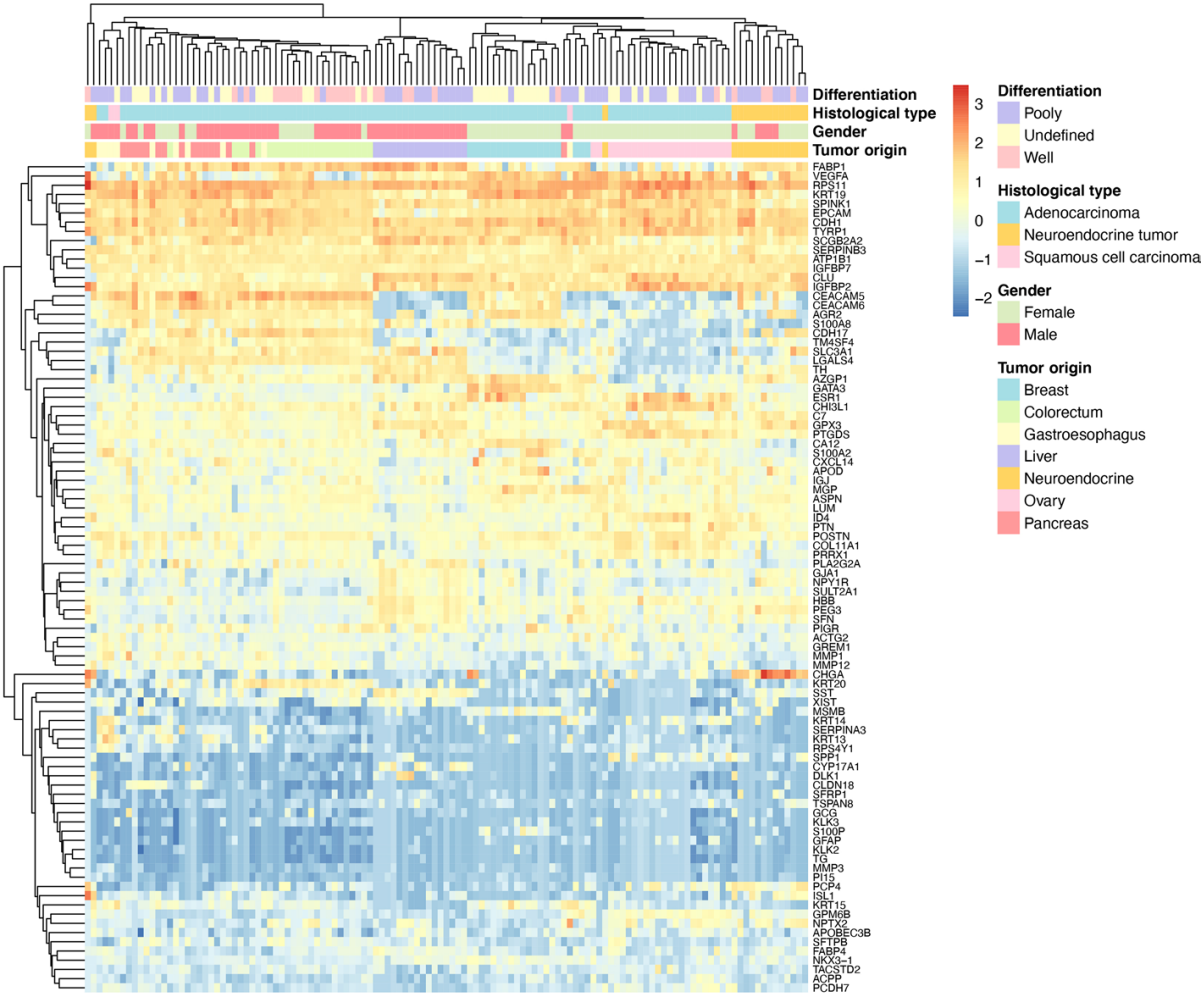
Hierarchical clustering analysis of 90 genes in 123 specimens. The average linkage hierarchical clustering method was performed where the metric of similarity was Pearson’s correlation between every pair of samples. The left panel shows a dendrogram of hierarchical clustering of 90 genes. Colored pixels capture the magnitude of the gene expression intensities, where shades of blue and red represent under-expression and over-expression, respectively, relative to the mean for each gene. The upper panel shows a dendrogram of hierarchical clustering of samples. The clinical features such as degree of differentiation, histological types, gender and tumor types of each sample are indicated in the upper panel. The number of tumor types less than five are not shown.

To illustrate the similarity between clinical samples, we performed hierarchical clustering based on primary liver cancer and six main metastatic tumor types (n >5). As shown in **Figure 3**, the samples were clustered into distinct groups that followed the tumor types based on the 90-gene expression pattern. The primary liver tumor samples were clustered together and showed distinct patterns from six LM types. Among LM types, digestive system neoplasms including colorectal, gastroesophageal, and pancreatic tumors were more likely to share similar gene expression patterns. For example, most of gastroesophageal tumors were clustered together, whereas few samples were similar to colorectal, pancreatic tumors.

A total of nine LM cases had discordant predictions compared with reference diagnoses. The histological types of nine misclassified samples included gastroesophageal (n=3), pancreas (n=2), lung (n=1), colorectum (n=1), neuroendocrine (n=1) and head&neck (n=1). Among nine cases, five were adenocarcinoma, three were squamous cell carcinoma and one was a neuroendocrine tumor. Eight of nine cases were poorly differentiated. The detailed characteristics of the discordant cases were investigated in **Table 3**.

**Table 3:** Investigation of nine cases misclassified by the 90-gene expression assay.

ID	Gender	Age	Sample type	Pathological diagnosis	90-gene expression assay predictions	Histological Subtype	Degree of differentiation
43	Male	69	Surgery	Gastroesophagus	Urinary	AC	Poorly
54	Male	61	Surgery	Colorectum	Gastroesophagus	AC	Well
57	Male	70	Biopsy	Pancreas	Colorectum	AC	Poorly
59	Male	74	Biopsy	Gastroesophagus	Liver	AC	Poorly
70	Male	53	Biopsy	Pancreas	Liver	AC	Poorly
75	Male	63	Biopsy	Lung	Head&neck	SCC	Poorly
114	Male	59	Surgery	Gastroesophagus	Liver	SCC	Poorly
119	Female	68	Surgery	Neuroendocrine	Endometrium	NET	Poorly
134	Female	63	Surgery	Head&neck	Liver	SCC	Poorly

AC, Adenocarcinoma; SCC, Squamous cell carcinoma; NET, Neuroendocrine tumor.

## Discussion

LM is the most common tumors encountered in the liver and continues to be a notable factor for morbidity and mortality. The identification of the primary tumor in the conditions of any LM is critical to define optimal management. In clinics, imaging modalities such as ultrasonography, CT, Magnetic resonance imaging (MRI), and positron emission tomography (PET) scans are typically most often applied for LM diagnosis (17). Hui et al. developed B-mode ultrasound radiomic models to distinguish the origin of liver metastatic lesions from the digestive tract tumor, lung tumor and breast tumor, with the sensitivity ranging from 70% to 75% (18). Moreover, serum tumor markers can potentially aid in the diagnosis of patients with LM. For instance, carcinoembryonic antigen (CEA) is one of the most crucial tumor markers for colorectal cancer. Other useful biomarkers for LM diagnosis include CA 19-9 (pancreaticobiliary cancer), chromogranin A (neuroendocrine tumor), CA 15-3 (breast cancer) and CA-125 (germinal tumor) (19). Although these serum markers are indicative for certain primary tumors, their specificities are still limited (19). For instance, the increase of serum CEA level may indicate the presence of colorectal cancer, but it can be also observed in 30-60% of pancreatic cancer patients (20).

Histological examinations including morphological and IHC analyses are the gold standard for tumor origin diagnosis. However, most of the LM originated from adenocarcinoma, which shares overlapping histological features with primary liver tumors or between each other (1). Thus, additional organ-specific IHC panels are crucial to characterize the tumor origin. A combination cytokeratin (CK) panel CK7/CK20 is recommended for initial evaluation (1, 8, 21). For example, CK7(-)/CK20(+) tumors may originate from colorectum, CK7(+)/CK20(+) tumors may originate from pancreas, biliary tract and gastroesophageal, etc., CK7(+)/CK20(-) tumors may originate from breast and ovary, etc., and CK7(-)/CK20(-) tumors may originate from hepatocellular carcinoma and squamous cell carcinoma (8, 21). However, many tumors express more than one phenotype, especially in gastrointestinal carcinoma. In a recent meta-analysis, IHC analysis correctly distinguished the primary site in 77.7% of metastatic liver cancers with the average usage of 6.9 ± 4.1 markers (8).

In recent years, several gene expression profiling-based assays were developed to identify the primary site of metastatic tumors. This technique is based on the theory that tumors share distinct gene expression patterns specific to their sites of origin (22). A commercial assay called CancerTYPE ID (Biotheranostics, San Diego, CA, USA), which is a RT-PCR assay involves 92 genes, allowing the identification of 28 common tumor types (23). A multisite validation study done by Sarah et al. demonstrates an overall sensitivity of 87% in primary site identification (24). Another assay named Tissue of Origin (TOO) test (Vyant Bio, New Jersey, USA) is microarray-based and measured the gene expression pattern of 1550 genes that related to 15 tumor types. In a multicenter cohort of 547 specimens, the TOO assay accurately classified 87.8% of cases (25). Over the past decades, DNA methylation profiling have been developed rapidly, which could be a useful approach to unmask the primary site of CUP. Sebastian et al. reported a DNA-methylation-based assay termed “EPICUP” for predicting primary sites of CUP (26). In a clinical validation set, EPICUP predicted a primary tumor of origin in 87% of CUP patients. More interestingly, patients with EPICUP diagnoses who received a tumor type-specific therapy showed improved overall survival compared with that in patients who received empiric therapy. However, neither of these assays has been validated in a large cohort of liver biopsy samples. Recently, only Katharina et al. reported a microRNA classifier showing an overall classification accuracy of 74.5% for primary site identification of liver biopsy specimens (27). This result was unsatisfactory for solving the urgent need of LM diagnosis in the clinic.

In the present study, the 90-gene expression assay achieved a precise classification of the tumor origin in 146 liver tumors with an overall accuracy of 93.8%, which was comparable to the EPICUP with 94% (501 of 534) accuracy in metastatic tumors (26). Moreover, the performance of the 90-gene expression assay was significantly better than the accuracy of the gold standard histopathology (77.7%) (8). In practice, the turnaround time of the 90-gene expression assay from archived FFPE samples to tumor type prediction was less than one day, which might greatly shorten patients’ waiting time compared with the conventional histopathological evaluation. These results indicated that the 90-gene expression assay might serve as a useful tool for accurately identifying the tissue of origin for liver tumors. In the daily diagnostic routine, FFPE liver biopsy specimens are widely used with limited amounts of tumor tissue and relatively high amounts of normal liver tissue than resections (27). Herein, this study enrolled 36 liver biopsy specimens, which were obtained by FNA or NCB. The analytic agreement reached 88.9% in biopsy specimens, which showed no statistically significant difference (p =0.31) with an accuracy of 95.5% in resection specimens. Therefore, this assay could be compatible with FFPE biopsy specimens, which allows widespread access and applications in clinical practice. However, we still noticed that nine cases were misclassified. As shown in **Table 3**, the most obvious of these cases relate to poorly differentiated tumors, which are likely more susceptible to deterioration of gene expression with increasing dedifferentiation. Given six of nine misclassified cases were gastrointestinal tumors (gastroesophageal, colorectum and pancreatic tumors), it could be argued that gastrointestinal tumors indeed shared more homogenous gene expression patterns compared with other tumor types, which was also observed in the unsupervised hierarchical clustering illustration (**Figure 3**).

Previous studies have shown that the gene expression patterns were sustained in LM compared to corresponding primary tumors, but normal liver tissue contamination in the surrounding must be considered as a potential cause of misclassification in gene expression analysis (27, 28). Katharina et al. reported a microRNA-based trained without contamination consideration showing a disappointing classification accuracy of 38.2%. By adjusting for liver contamination, the classifier’s accuracy was significantly improved to 67.3% (27). In the present study, we set high tumor content criteria above 60% to minimize possible confounding factors. Indeed, the overall accuracy of the 90-gene expression assay showed significant improvement compared with Katharina et al’s results. However, we still observed four cases (two gastroesophageal cancer, one pancreatic cancer, and one head&neck tumor) were misclassified as primary liver cancer, suggesting the consistent source of variation induced by various tumor contents and infiltrating immune cells in the tumor environment would impact the gene expression analysis results. It’s worth noting that although selecting high tumor content samples would be helpful to reduce the normal liver tissue contamination, this might significantly restrict the utilization of the gene expression assay in real clinical setting.

Besides, the present study also had several other limitations. First, the performance of the 90-gene expression assay is variable across different tumor types due to small enrolled number of certain tumor types. For example, the sensitivity is ranged from 50% (head&neck) to 100% (ovary, breast, melanoma, etc.). Further validation of the 90-gene expression assay on larger numbers of head&neck origin LM, gastroesophageal origin LM, rare LM types, and poorly differentiated LM are warranted. Second, although the 90-gene expression assay demonstrated an accuracy of 100% in classifying the neuroendocrine tumors from various origins, however, it was unable to evaluate the discriminating performance of the panel to distinguish the tumor origins of neuroendocrine tumors.

## Conclusion

In conclusion, the results of the present study demonstrate encouraging performance of the 90-gene expression assay for distinguishing primary liver tumor from LM and identifying the primary sites of LM. In cases that morphology and IHC analyses cannot confirm the tissue of origin, the 90-gene expression assay maybe serves as a helpful instrument for discriminating the primary tumor. Future incorporation of the 90-gene expression assay in clinical diagnosis will aid oncologists in applying precise treatments, leading to improved care and outcomes for LM patients. In future studies, additional effort needs to be done for the distinguishing of head&neck origin LM, gastroesophageal origin, rare LM types, or poorly differentiated LM.

## Data Availability Statement

The original contributions presented in the study are included in the article/**Supplementary Material**. Further inquiries can be directed to the corresponding authors.

## Ethics Statement

The studies involving human participants were reviewed and approved by the institutional review board of Fudan University Shanghai Cancer Center (FUSCC, Shanghai, China), West China Hospital Sichuan University (WCHSU, Chengdu, Sichuan, China), Sichuan Cancer Hospital (SCH, Chengdu, Sichuan, China), Chongqing Cancer Hospital (CCH, Chongqing, China), Tianjin Medical University Cancer Institute & Hospital (TMUCIH, Tianjin, China), Sir Run Run Shaw Hospital (SRRSH, Hangzhou, Zhejiang, China) and Hubei Cancer Hospital (HCH, Wuhan, Hubei, China). Written informed consent to participate in this study was provided by the participants’ legal guardian/next of kin.

## Author Contributions

XZ, WL, BM, and JY designed the study. QW, FL, QJ, QL, HA, YL, ZL, LF, FG, and BM provided the specimens and collected clinical information. YS, YW, and WR performed the experiments. QW, YS, and QX analyzed all data. QW and YS wrote the initial manuscript draft. XZ, WL, BM, JY, and QX critically revised the manuscript and gave valuable insight to the study concept. All authors contributed to the article and approved the submitted version.

## Funding

This work was partially supported by research funding from Innovation Program of Shanghai Science and Technology Committee grants 20Z11900300 (XZ), Innovation Group Project of Shanghai Municipal Health Commission grants 2019CXJQ03 (XZ), Shanghai Science and Technology Development Fund grants 19MC1911000 (XZ, QW) and Shanghai Municipal Key Clinical Specialty grants shslczdzk01301 (XZ, QW), the National Natural Science Foundation of China grants 81,401,963 (QW) and 81,972,728 (QW), Fudan University Shanghai Cancer Center grant YJYQ201603 (QW), Health commission of Hubei Province scientific research project grant WJ2019H124 (JY), Hubei science and technology program grant 2020CFB869 (FG) and Canhelp Genomics Co., Ltd.

## Conflict of Interest

Author YS, YW, WR, and QX were employed by the company Canhelp Genomics.

The remaining authors declare that the research was conducted in the absence of any commercial or financial relationships that could be construed as a potential conflict of interest.

## Publisher’s Note

All claims expressed in this article are solely those of the authors and do not necessarily represent those of their affiliated organizations, or those of the publisher, the editors and the reviewers. Any product that may be evaluated in this article, or claim that may be made by its manufacturer, is not guaranteed or endorsed by the publisher.
