# Supplementary material for: Gene Expression Profiling for Differential Diagnosis of Liver Metastases: A Multicenter, Retrospective Cohort Study
Source: Front Oncol. 2021 Sep 22;11:725988. doi: 10.3389/fonc.2021.725988 (PMC8493028; doi:10.3389/fonc.2021.725988)
Supplement: Supplementary file 1 [file DataSheet_1.docx]

Supplementary Material

# Supplementary Figures and Tables

## Supplementary Figures


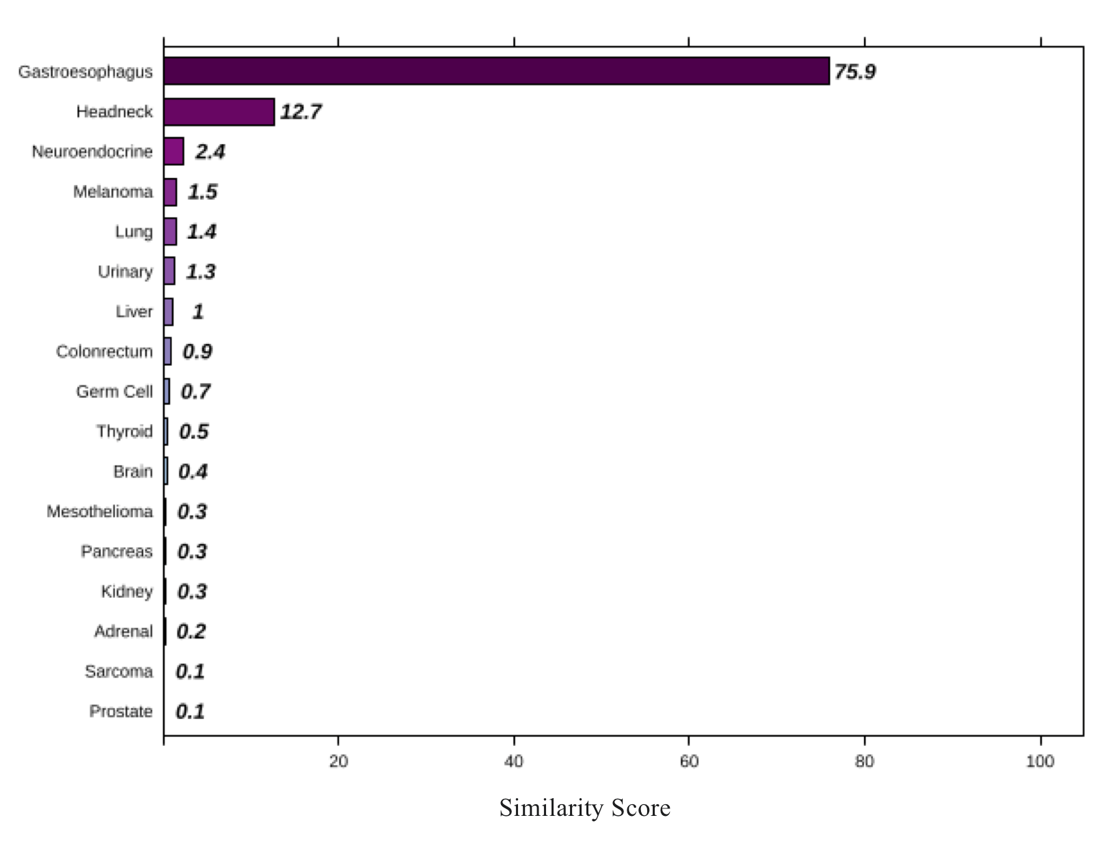


**Supplementary Figure 1.** An example of the 90-gene expression assay result. Gene expression pattern was analyzed with the 90-gene classifier, with one similarity score for each of the 21 tumor types. The top five tissue origin with the highest similarity scores are as follows: Gastroesophagus (75.9), Head and neck (12.7), Neuroendocrine (2.4), Melanoma (1.5), Lung (1.4), suggesting the most likely tissue of origin is Gastroesophagus (75.9).

## Supplementary Tables

**Supplementary Table 1.** List of 21 tumor types predicted by the 90-gene expression assay. Names of the tumor types included in the present study were bolded and underlined.

| Number | Tumor types |
| --- | --- |
| 1 | Adrenal |
| 2 | Brain |
| 3 | **Breast** |
| 4 | **Cervix** |
| 5 | **Colorectum** |
| 6 | Endometrium |
| 7 | **Germ cell** |
| 8 | **Gastroesophagus** |
| 9 | **Head and neck** |
| 10 | **Kidney** |
| 11 | Liver |
| 12 | **Lung** |
| 13 | **Melanoma** |
| 14 | Mesothelioma |
| 15 | **Neuroendocrine** |
| 16 | **Ovary** |
| 17 | **Pancreas** |
| 18 | Prostate |
| 19 | **Sarcoma** |
| 20 | Thyroid |
| 21 | **Urinary** |

**Supplementary Table 4.** Sample enrollment of seven hospitals.

Abbreviation: FUSCC, Fudan University Shanghai Cancer Center; WCHSU, West China Hospital Sichuan University; SCH, Sichuan Cancer Hospital; CCH, Chongqing Cancer Hospital; TMUCIH, Tianjin Medical University Cancer Institute & Hospital; HCH, Hubei Cancer Hospital; SRRSH, Sir Run Run Shaw Hospital

| **Center** | Liver | Ovary | Colorectum | Breast | Neuroendocrine | Pancreas | Gastroesophagus | Melanoma | Cervix | Lung | Adrenal | Germ Cell | Head and Neck | Sarcoma | Kidney | Urinary | Total |
| --- | --- | --- | --- | --- | --- | --- | --- | --- | --- | --- | --- | --- | --- | --- | --- | --- | --- |
| FUSCC | 16 | 7 | 22 | 7 | 5 | 10 | 7 | 2 | 1 | 3 | 2 | 1 | 1 | 2 | 1 | / | 87 |
| WCHSU | / | 4 | / | 4 | 2 | 6 | 2 | / | / | / | 1 | / | / | / | / | / | 19 |
| SCH | / | 2 | / | 4 | / | / | / | 2 | 3 | / | / | / | / | / | 1 | / | 12 |
| CCH | / | 5 | / | 1 | 2 | / | / | / | / | / | / | 1 | 1 | / | / | / | 10 |
| TMUCIH | / | 1 | / | 4 | 3 | / | / | / | / | / | / | / | / | / | / | / | 8 |
| SRRSH | / | 3 | / | / | 1 | / | 1 | / | / | / | / | / | / | / | / | 1 | 6 |
| HCH | / | 1 | / | / | 3 | / | / | / | / | / | / | / | / | / | / | / | 4 |
| Total | 16 | 23 | 22 | 20 | 16 | 16 | 10 | 4 | 4 | 3 | 3 | 2 | 2 | 2 | 2 | 1 | 146 |
